# Why and When Bacterial Vaccines Should Be Given

**Published:** 1913-09

**Authors:** W. Frank Wells

**Affiliations:** Atlanta, Ga.


### WHY AND WHEN BACTERIAL VACCINES SHOULD BE GIVEN.

By W. Frank Wells, M. D., Atlanta, Ga.

Immunity is based upon the following principle: Every living organism, both animal and vegetable, possesses the property of defending itself against other living organisms. This power is based upon the innate power of these organisms to defend themselves by building up within themselves defensive products. These products have been named by serologists, "antibodies." Time will not permit of a complete description of each of these antibodies, and suffice it to say that they consist of substances which destroy the attacking organisms itself, and also, substances which destroy the defensive products built up by these attacking organisms. The antibodies which destroy the defensive products of these organisms are called antitoxins, and antibodies which destroy the organisms are called lysins. This word is from the Greek word "Luo" which means to disintegrate or loosen. They consist of bacterins, cytolysins, hemolysins, protolysins, etc. These substances act by a process of digestion very similar to that carried on in the stomach in digesting a piece of beef. As stated in the outset, these bodies exist normally in every living structure, and just as every living structure is stimulated to a greater activity by acting, just so are these bodies. Therefore, the process of immunity is brought about by the stimulous placed upon these bodies from the substance entering from without, upon which the above antibodies by nature are trained to act. After these have received sufficient training to protect their host against the specific invading organisms, then that host is said to be immune against this organism.

Should these organisms and their defensive products (toxines) be so powerful as to cripple and maim the cell structures of the body to the degree that defensive products in the form of antibodies cannot be built up sufficiently to combat them, the result is not immunity, but a projection of the cell structures ensues, and death.

After virulent germs have kept up invasive process in the body structures for a long period of time, when no immunity is established nor death ensues from the invasion, there results a condition which we might term a compromise between the tissues and the invading organisms. In other words, there is a 'flag of truce' established, neither one being able to whip the other. This is where the art comes in to aid nature. The cell structures cannot build up antibodies sufficiently antagonistic to destroy the bacteria and their metabolic products, so they fight indefinitely. What is needed now is some stimulous that will arouse a greater activity on the part of these cells. Any substance that will arouse this activity is called an antigen, which word means a product that will stimulate the building up of antibodies. The virulent bacteria themselves are antigen until the above named 'flag of truce' is declared, but after this becomes so we must insert into the tissues an artificial antigen.

We obtain this from a specific germ causing the disease, by attenuating either the germ itself or its metabolic product. This attenuation is brought about by a multiplicity of ways; the germs may be heated to 60 degrees for one hour; they may be ground until they become thoroughly disintegrated; they may be grown at a temperature higher than the body; they may be grown upon a laboratory culture media until they in a measure lose their virulence, or they may be grown in some animal with greater resisting power than the suffering individual; or their metabolic products may be filtered out from these germs after they have been attenuated by these various methods. They may also be attenuated by drying.

Cell structures which have lost their power to combat the virulent germs will be able to attack these attenuated substances. Therefore, when they are inserted into the tissues in small quantities the tissues will attack these products successfully. Then as larger quantities are introduced more and stronger antibodies are built up and they are trained more successfully to attack them. This process is continued by gradually increasing doses. These dosages serving as antigen until the antibodies are sufficiently trained to attack the more virulent tribes, which are invading the tissues producing the disease, thereby establishing an immunity by training up cells which had previously been in a state of lethargy.

The question arises in the minds of many men who have not made a special study of immunity as to the difference in the various substances that are used in this process. We have to contend with such names as vaccines, bacterins, serums, antigens, phylacogens, etc. We need an improvement in our nomenclature and place them all under the heads of serums and antigens. The difference between the serum and antigen is that the serum is a product that produces a passive immunity by injecting antibodies which have been built up in another animal. An antigen is either attenuated bacteria themselves or their metabolic products which are injected into the tissues and which train those tissues to build up specific antibodies to destroy the specific germ (active immunity). Bacterins, which are simply bacteria killed by heating to 60 degrees for one hour, vaccines produced in the same way and phylacogens produced by obtaining metabolic products or the bacteria, are all antigens, and all in scientific discussions should be called by this name in order to avoid confusion. In fact, the word vaccine has crept into our medical nomenclature on account of the fact that small pox was the first disease cured by this process and it was done by attenuating them in a cow, and the word vacca meaning a cow. Our process of introducing attenuated bacteria is so similar to the process of vaccination in small pox that we now use the term vaccine in a general way to apply to all methods of producing active immunity.

From the foregoing conclusions I will now state when bacterial vaccines should be administered. They should be given to render a person immune from a disease or they should be given to aid nature in building up antibodies in the blood. The time to give vaccines in disease is not at the height of the disease, when the temperature is very high and the patient septic. Should it be given then it would be of no value as the cells are already poisoned and crippled to such an extent that stimulating them would be of no good. For example, if you have driven a horse until there is no life in him, he is simply exhausted and can run no more, whipping him would be of no value. But if you stop him and rest him and feed him up a little, then apply the whip it is of some value. So bacterins should be given before the disease becomes acute or after the above stated 'flag of truce' has been established.

If you have a known infection that has gotten into the system in some way, such as hydrofobia, then before the disease has established itself you can administer the bacterins and build up an immunity against it; or you may have a Strepto Coccic infection in the hand from a Septic operation or a post-mortem, and see that the infection is making it's way into the system by the red streaks up the arm, and the patient becoming somewhat nauseated with a slight chill, then is the time to give the Strepto Bacterins. If you wait until the entire system is saturated with the toxins from the infection and the cells are doing all they can, then it would be useless to stimulate them to do more.

The time to give bacterins in typhoid fever is not when the patient has been sick long enough to become septic and rose spots showing up and a dark brown tongue with much distention of the bowels. At such a time the bacterin treatment will be of no value whatever. The same is true in tuberculosis. But wait until the fever has been lowered by other methods and the septic condition becomes better, then if the patient improves slowly and the disease still lingers and the 'flag of truce' is established, then give the bacterins beginning with the proper doses. Every patient is a law unto himself as to what the dose should be and how to increase the dose.

The treatment of disease by the use of serums and antigens is coming to be more and more used in the medical world. After all it is but a stimulation of nature, causing her to use her own best methods of defending the body cells against deadly germs. The time is not far hence when every physician will not only appreciate the value of serums and antigens, but will be using them freely and intelligently in the course of his practice.

